# Superhydrophobic Polytetrafluoroethylene/Heat-Shrinkable Polyvinyl Chloride Composite Film with Super Anti-Icing Property

**DOI:** 10.3390/polym11050805

**Published:** 2019-05-06

**Authors:** Zhiqing Jiang, Xueqin Wang, Huiying Jia, Yanfen Zhou, Jianwei Ma, Xinyu Liu, Liang Jiang, Shaojuan Chen

**Affiliations:** College of Textiles and Clothing, Qingdao University, Qingdao 266071, China; jiang.zhiqing@outlook.com (Z.J.); jia.huiying@outlook.com (H.J.); yanfen.zhou@outlook.com (Y.Z.); mjwfz@hotmail.com (J.M.); jaystarrain@163.com (X.L.); liang.jiang@outlook.com (L.J.)

**Keywords:** polytetrafluoroethylene/heat-shrinkable polyvinyl chloride composite film, vacuum thermal evaporation, superhydrophobicity, anti-ultraviolet, anti-icing

## Abstract

Wind power generation is an environmentally friendly way to produce electricity, but wind turbine blades that are prone to freeze in winter will reduce the efficiency of the generator. Therefore, the preparation of anti-icing blades is important and essential. Herein, anti-icing polytetrafluoroethylene (PTFE)/heat-shrinkable polyvinyl chloride (HSPVC) composite film was prepared by depositing a PTFE coating on the surface of HSPVC film via vacuum thermal evaporation. HSPVC films were pretreated respectively by argon and carbon tetrafluoride (CF_4_) plasma cleaning to introduce new groups and change their surface energy. After that, PTFE coating with a thickness of about 4 μm was deposited on the surface of HSPVC, obtaining a superhydrophobic surface with an apparent water contact angle of 150°. The results demonstrated that the breaking strength of the PTFE/HSPVC composite film using CF_4_ plasma pretreatment decreased by only 3.47% after exposing to ultraviolet light with the power of 1000 W for 5 min, suggesting an excellent anti-ultraviolet property. Furthermore, compared with the pristine films, the PTFE/HSPVC composite films exhibited better adhesive strength, super anti-icing property even after 10 icing–deicing cycles, and excellent dynamic anti-icing performance. The PTFE/HSPVC composite film with good adhesive strength, anti-ultraviolet, and anti-icing properties has prospective applications in packaging of wind turbine blades.

## 1. Introduction

Wind turbines are widely used to produce clean electricity, but their blades tend to freeze in frosty weather, which increases the weight of the blades, reduces their efficiency, damages the machine, and can threaten pedestrian safety. Many efforts have been made to prevent wind turbine blades from freezing, such as manual deicing, mechanical deicing, and thermal deicing. However, manual deicing wastes manpower and material resources, mechanical deicing damages equipment, and thermal deicing wastes energy by energizing the resistance wires [1,2]. A heat-shrinkage film with superhydrophobic and anti-ultraviolet (UV) properties can be used to coat the blades of wind turbines, thus avoiding the adhesion between frost and blades. Heat-shrinkable polyvinyl chloride (HSPVC) film has been widely used in various fields such as building materials and packaging industry [3,4], owing to its unique shrinkable properties, high transparency, excellent glossiness, strong mechanical strength, and outstanding self-stickiness [5]. However, the poor UV resistance of the HSPVC films has severely limited their practical applications in long-term outdoor coatings. Moreover, HSPVC is a hydrophilic film, which promotes adhesion between itself and frost [6].

In order to improve the hydrophobicity and UV resistance of the HSPVC film, surface modification of HSPVC film is normally required. Improving the hydrophobicity of the material surface is an effective way to delay or prevent freezing [7]. The general process for preparing a superhydrophobic surface is the construction of surface roughness and modification with low-surface-energy coatings [8]. Widely used methods for the surface modification of materials include radiation grafting, [9,10,11], electrospinning [12], sol-gel method [13,14], magnetron sputtering [15,16,17], self-assembly [18], and vacuum thermal evaporation. Kim et al. [19] synthesized fluorosilicone copolymers by free-radical random copolymerization using fluorine-containing monomers and silicon-containing monomers as raw materials to improve the hydrophobicity of PVC and increase the apparent water contact angle (AWCA) [20,21] of PVC to 117°. Hydrophobic coatings can adhere to the PVC surface to change the surface properties of PVC, but the ideal hydrophobic anti-icing effect cannot be obtained. In order to obtain both anti-UV and hydrophobic properties of HSPVC film, the choice of coating materials should be emphasized. Polytetrafluoroethylene (PTFE) with a fluorinated alkyl radical is well known for its chemical inertness, low surface energy, good hydrophobicity, and excellent anti-UV property [22,23,24], and is frequently used to improve the surface hydrophobicity of other materials [25,26,27,28,29]. There are some reports on the performance of PTFE coating deposited on the surface of glass substrates using vacuum thermal evaporation technique. For example, Yi et al. [30] fabricated a three-dimensional porous network PTFE thin film with an AWCA of 155° on glass by thermal deposition of PTFE powders. Wang et al. [31] prepared a superhydrophobic PTFE coating for microelectronics, buildings, and biotechnology by depositing PTFE nanoparticles on the glass via vacuum thermal evaporation. However, the effect of PTFE coating deposited on the surface of flexible material (such as HSPVC film) on the performance of substrate has rarely been systematically studied.

In this paper, we fabricated a transparent super anti-icing PTFE/HSPVC composite film as a packaging material for wind turbine blades using the vacuum thermal evaporation method. HSPVC films were respectively pretreated by plasma cleaning under the atmosphere of argon (Ar) and carbon tetrafluoride (CF_4_), and then PTFE powders were evaporated to deposit on HSPVC film. The surface micromorphology and chemical structure of PTFE coatings deposited under different pretreatment conditions were studied. The AWCA and the changes in AWCA were measured to verify the hydrophobicity and adhesive strength of PTFE coatings. Furthermore, study of the anti-UV and anti-icing properties was carried out to confirm the possibility of the modified PTFE/HSPVC composite film for outdoor packaging of wind turbine blades.

## 2. Experimental

### 2.1. Materials

PTFE powders were purchased from Macklin, Shanghai, China. HSPVC films were supplied by Wuxi Kailian Special Materials Co., Ltd., Wuyi, China.

### 2.2. Sample Preparation

**Figure 1:**
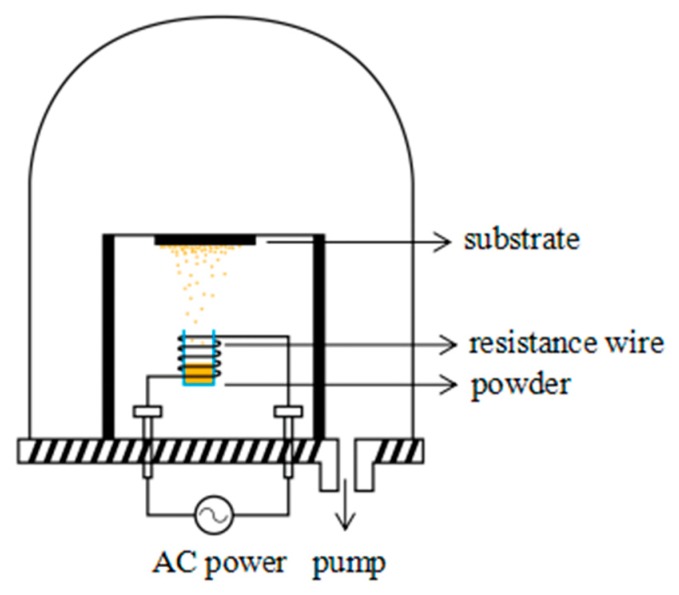
Schematic description of the vacuum thermal evaporation system.

PTFE-coated HSPVC films were prepared using a vacuum thermal evaporation apparatus (ZHD-300M2, Technol Science Co., Ltd., Beijing, China) and the working principle of the system is schematically described in Figure 1. Before the deposition, the HSPVC films were respectively cleaned with alcohol and deionized water under ultrasonic agitation for 1 h to remove impurities, and then dried in a vacuum oven. Subsequently, the specimens were divided into three groups. The first group was not pretreated by plasma gas and named M_1_. The second and last group were treated separately using Ar and CF_4_ plasma cleaning at a flow rate of 50 standard cubic centimeters/min (sccm) under a direct-current (DC) power of 50 W for 5 min and named M_2_ and M_3_, respectively. Furthermore, all specimens were deposited with PTFE nanoparticles in the vacuum evaporation system using the same evaporation process at room temperature. The samples were named respectively as M_1_-1, M_2_-1, and M_3_-1. The crucible with 0.2 g PTFE powders was heated to 380 °C by adjusting the current of the resistance wire to 28 A. The evaporation time was set to 5 min in high vacuum of about 5.0 × 10^−3^ Pa. Finally, all modified films were separately treated with shrinkage for 30 s and 60 s at 70 °C. After shrinking for 30 s, the modified films were named M_1_-2, M_2_-2, and M_3_-2, respectively, and they were correspondingly named M_1_-3, M_2_-3, and M_3_-3 when they were shrunk for 60 s.

### 2.3. Anti-Icing Testing of Samples

The static and dynamic anti-icing property of shrinking pristine HSPVC film and shrinking PTFE/HSPVC composite films were observed in a self-made artificial climate chamber. Water droplets at 3 °C were dripped on the surface of the samples with a speed of 5 L/h and the size of the water droplets was about 50 μL. In order to test the static anti-icing property, the samples were vertically fixed in the climatic chamber and the inner ambient temperature was set to −5 °C to freeze for 30 min. After calculating the freezing area ratio of each sample, the ice is removed immediately, and then those samples are put into the artificial climate chamber for the next freezing (freezing area is calculated by Adobe Photoshop CS6). This cycle was repeated 10 times. Meanwhile, the dynamic anti-icing performance of the composite film was simulated by an electric fan (Deli, Zhejiang, China). The electric fan with rotational speed of 180 rpm was placed in an artificial climate chamber at −5 °C for 3 h. During this process, the water droplets (size of 50 μL and temperature of 3 °C) would continuously drop onto the surface of fans (including the samples) with a speed of 5 L/h. After 3 h, the electric fan was removed from the artificial climate chamber, and the freezing morphology of the samples was observed and photographed.

### 2.4. Characterization

Surface morphologies of the modified films were observed by using scanning electron microscopy (SEM, Phenom, Eindhoven, Netherlands) and atomic force microscopy (AFM, CSPM 5500, Being Nano, Beijing, China). The coverage rate of PTFE nanoparticles on HSPVC was calculated by Adobe Photoshop CS6. The coverage area refers to the area where the PTFE nanoparticles are combined with the surface of HSPVC. The mapping of F element was measured using energy dispersive spectrometer (EDS, Genesis Apex, EDAX, Movo, New Jersey, America). The surface chemical compositions of the specimens were determined by using X-ray photoelectron spectroscopy (XPS, Thermo Scientific Escalab 250Xi, Thermo Fisher Scientific, Philon, New Jersey, America). The AWCA of modified films were measured using optical contact angle meter (XG-CAMB1, Shanghai Xuanyichuanxi, Shanghai, China) to evaluate their hydrophobicity. A piece of 1 × 1 cm^2^ sample was attached onto a specimen stage and 2.0 μL deionized water was dropped onto the surface of the sample. The contact angle hysteresis (CAH) of water droplet on the surface of sample is obtained by using automatic tilting plate. The surface energy of the sample was automatically calculated using the Zisman Plot program on the optical contact angle meter by adding two different polar liquids, distilled water and diiodomethane, to the surface of the sample. A universal strength testing machine (YG026H, Wenzhou Darong, Wenzhou, China) was employed to measure the breaking strength of the contractive modified HSPVC films after exposing for 1 to 5 min under ultraviolet lamp (1000 W, Osram, Munich, Germany) to indirectly characterize their UV resistance. The size of the samples to be tested is 2 cm × 10 cm. All PTFE/HSPVC composite films after shrinking were bonded with copper foil adhesive tapes under the force of 10 N and subsequently stripped at the same speed. The process was repeated 5 times and the changes of AWCA were recorded correspondingly. The adhesive strength between HSPVC film and PTFE coating was indirectly proved by exploring the changes in AWCA. 

## 3. Results and Discussion

### 3.1. Surface Morphology

**Figure 2:**
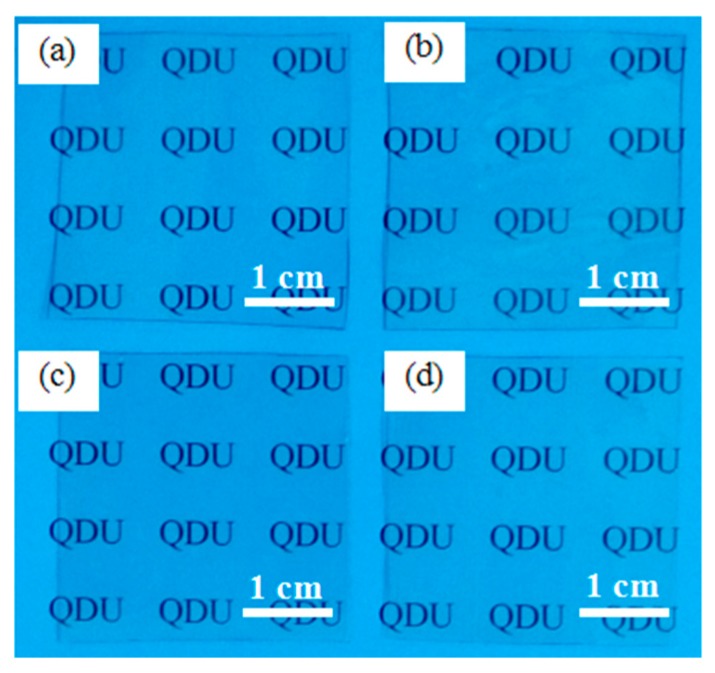
The digital photos of (**a**) shrinking pristine film, (**b**) M_1_-1, (**c**) M_2_-1, and (**d**) M_3_-1.

**Figure 3:**
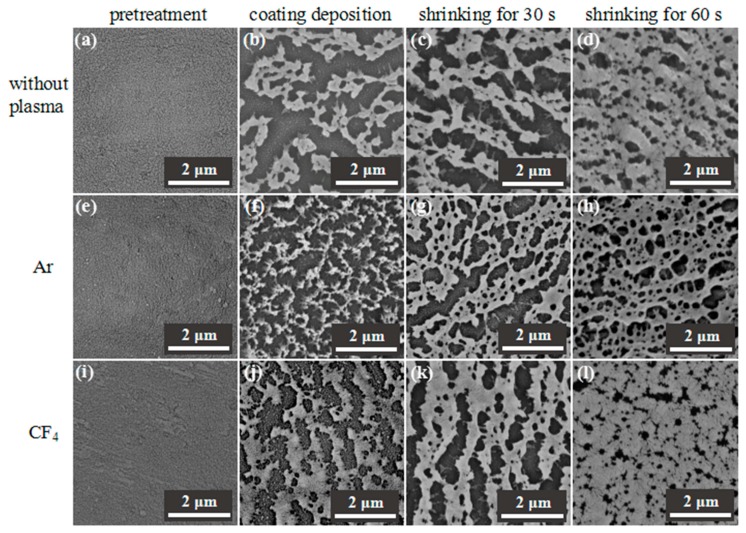
SEM images of heat-shrinkable polyvinyl chloride (HSPVC) films after pretreatment, coating deposition, and surface shrinkage. (**a**) M_1_, (**b**) M_1_-1, (**c**) M_1_-2, (**d**) M_1_-3, (**e**) M_2_, (**f**) M_2_-1, (**g**) M_2_-2, (**h**) M_2_-3, (**i**) M_3_, (**j**) M_3_-1, (**k**) M_3_-2, (**l**) M_3_-3.

The transparent PTFE/HSPVC composite films prepared by vacuum evaporation technology are shown in Figure 2. Compared with the original HSPVC, the modified films have no obvious difference in appearance, and the characteristics of M_1_-1, M_2_-1, and M_3_-1 can be clearly seen. The surface micromorphology and structure of the films are important factors affecting the construction of superhydrophobic surfaces [32]. In order to evaluate the structure and uniformity of the PTFE coating, the surface morphology of modified HSPVC films before and after shrinking was observed by SEM and the corresponding images are shown in Figure 3. The surface morphology of the HSPVC films was slightly changed by plasma treatment under the atmosphere of Ar and CF_4_. When the PTFE powders were evaporated, the PTFE nanoparticles on each surface of film exhibited a Volmer–Weber mode. However, the growth morphology of PTFE nanoparticles deposited on HSPVC films is not entirely consistent due to the different surface energy of HSPVC films pretreated with different methods. The surface energy of the pristine HSPVC film is small, and when the PTFE nanoparticles are deposited, the bonding between the PTFE atoms is likely to occur, so the uniformity of the PTFE nanoparticles deposited on the pristine HSPVC film is relatively poor. After Ar plasma cleaning, the surface of the HSPVC film was activated and the number of active groups was increased [33]. Meanwhile, the surface energy of the film was increased, and the morphology of PTFE nanoparticles on the HSPVC film became more uniform. Although the surface energy of the HSPVC film decreases based on the CF_4_ plasma pretreatment, the introduction of fluorine (F) element originating from CF_4_ plasma cleaning might provide favorable conditions for the subsequent spread of the island. Therefore, the results through calculations show that the fraction of coverage of PTFE nanoparticles on M_1_-1, M_2_-1, and M_3_-1 was 37.37%, 35.7%, and 55.29%, respectively, which might affect the AWCA of the PTFE coatings. It can be speculated that CF_4_ plasma cleaning promotes the deposition of PTFE nanoparticles on HSPVC films. After heating for 30 s, the PTFE nanoparticles on the surface of deposited modified HSPVC films gradually approached uniformity along with the shrinkage of HSPVC films. After heating for 60 s, all of the samples showed a microporous morphology. However, the pores of M_3_-3 are relatively small compared with others after heating for the same time due to the high coverage rate of PTFE nanoparticles on the surface of M_3_-1.

**Figure 4:**
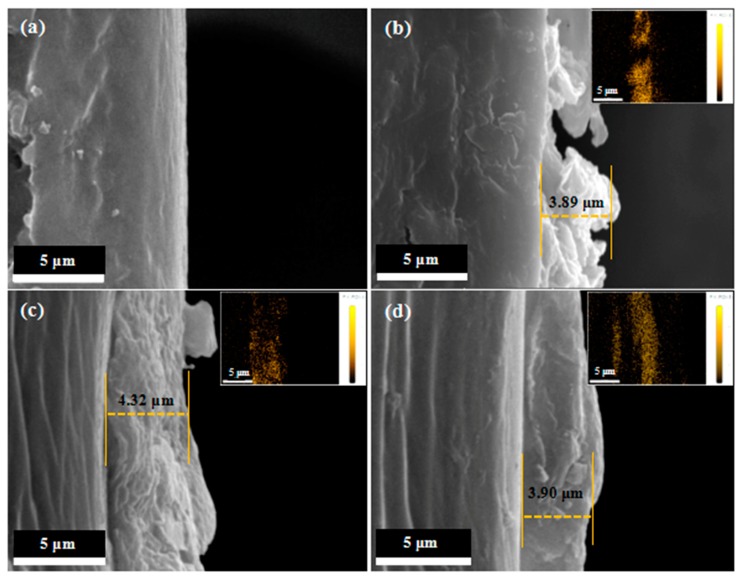
SEM images represent the cross sections of (**a**) pristine film, (**b**) M_1_-2, (**c**) M_2_-2, and (**d**) M_3_-2. The illustrations correspond to the mapping photos of F element of (**b**) M_1_-2, (**c**) M_2_-2, and (**d**) M_3_-2.

The cross sections of pristine film and PTFE/HSPVC composite films were used to measure the thickness of the PTFE coatings and the formation of the PTFE coatings was confirmed by the mapping of F element. As can be seen from Figure 4, the thickness of PTFE coating deposited on the surface of HSPVC was 4.32 μm using Ar plasma pretreatment, while the formed PTFE coatings using CF_4_ plasma pretreatment or no plasma pretreatment exhibited a smaller thickness. In addition, compared with M_1_-2, PTFE nanoparticles deposited by vacuum thermal evaporation of PTFE powders after plasma cleaning are denser. The morphology and the thickness of the PTFE coatings on the cross section of modified HSPVC are described by the mapping of F element.

**Figure 5:**
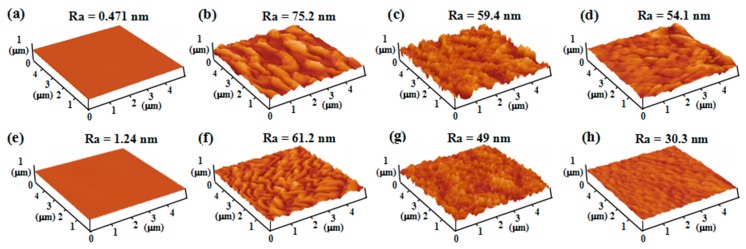
Atomic force miscroscopy (AFM) images of (**a**) pristine film, (**b**) M_1_-1, (**c**) M_2_-1, (**d**) M_3_-1, (**e**) pristine film after shrinking for 60 s, (**f**) M_1_-3, (**g**) M_2_-3, (**h**) M_3_-3.

Figure 5 shows AFM images for pristine film, PTFE/HSPVC composite films, and shrinking PTFE/HSPVC composite films. The surface of the original HSPVC film is smooth and uniform and there is no significant difference in the surface morphology of the samples before and after shrinking. Under the same evaporation process, different surface morphologies were formed on the surfaces of M_1_-1, M_2_-1, and M_3_-1, revealing that the surface properties of the films were changed under different pretreatment methods. This phenomenon is ascribed to changing the polar component of the surface energy after Ar or CF_4_ plasma pretreatment [34]. After shrinking for 60 s, the PTFE nanoparticles on the surface of the HSPVC films approached with the contraction of the substrates, forming denser PTFE coatings. This also verifies the conclusion of the SEM images.

### 3.2. Chemical Composition of PTFE Coatings

**Figure 6:**
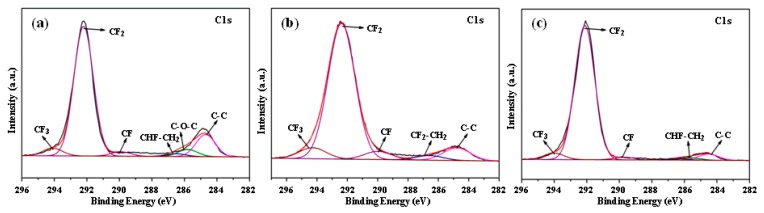
XPS C1s spectra of the deposited modified PVC films pretreated with (**a**) nonplasma cleaning, (**b**) Ar plasma cleaning, and (**c**) CF_4_ plasma cleaning.

The chemical composition of the material significantly affects its physicochemical properties, such as surface wettability and UV resistance [35,36]. Figure 6 shows the XPS C1s spectra of PTFE coatings obtained by vacuum thermal evaporation with different pretreatment methods. All deposited PTFE coatings exhibit one strong peak corresponding to -CF_2_ and two weak peaks corresponding to -CF and -CF_3_, respectively. Contents of F element and C element in the PTFE coatings with different pretreatment methods are shown in Table 1. As can be seen from Table 1, PTFE coatings prepared by evaporation of PTFE powders after plasma pretreatment showed an F/C ratio greater than that of PTFE. Deposition on the HSPVC film using CF_4_ plasma cleaning exhibited the highest F/C elemental ratio. This might have resulted from the introduction of some F element on the surface of HSPVC film by CF_4_ plasma pretreatment.

### 3.3. AWCA of Modified HSPVC Films

**Figure 7:**
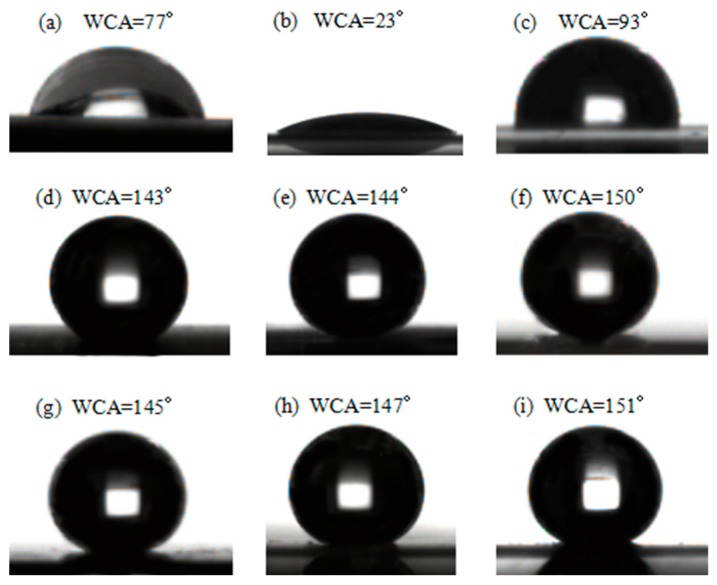
Apparent water contact angle (AWCA) images of (**a**) M_1_, (**b**) M_2_, (**c**) M_3_, (**d**) M_1_-1, (**e**) M_2_-1, (**f**) M_3_-1, (**g**) M_1_-3, (**h**) M_2_-3, (**i**) M_3_-3.

Changes in the AWCA of the HSPVC films under different modification methods are shown in Figure 7. It was found that the AWCA of the pristine HSPVC film was 77°, and its surface energy was 36 mN/m. Compared with pristine HSPVC film, the AWCA of M_2_ decreased significantly to 23°, while its surface energy increased to 51.8 mN/m after Ar plasma cleaning for 5 min. This might be due to the erosion of Ar, which destroys the surface structure of the HSPVC film and introduces new hydrophilic groups [37,38,39]. After pretreatment with CF_4_ plasma, the AWCA of the HSPVC film increased to 93°, and its surface energy decreased to 32.7 mN/m. This phenomenon might be ascribed to the erosion of CF_4_ plasma and the introduction of F element. Ions generated on the surface of substrate by the glow discharge can destroy the surface structure of the substrate, resulting in the formation of fluorinated groups [40,41,42,43]. Changes in the surface energy of the HSPVC under different pretreatments might explain the formation of different micromorphology of the PTFE coatings in Figure 3. When PTFE nanoparticles were deposited on HSPVC films, the AWCA of the three films increased greatly, but only the AWCA of M_3_-1 exceeded 150°, resulting in a superhydrophobic effect. This behavior might have resulted from fluorinated groups being easily introduced into the surface of the substrate after CF_4_ plasma pretreatment [44]. After shrinking for 60 s, the AWCA of all films increased slightly. After automatically tilting the sample table, it was found that M_3_-3 had a low CAH of water droplet (~4°), and the CAH of M_1_-3 and M_2_-3 were both about 6°.

### 3.4. UV Resistance of PTFE/HSPVC Composite Films

**Figure 8:**
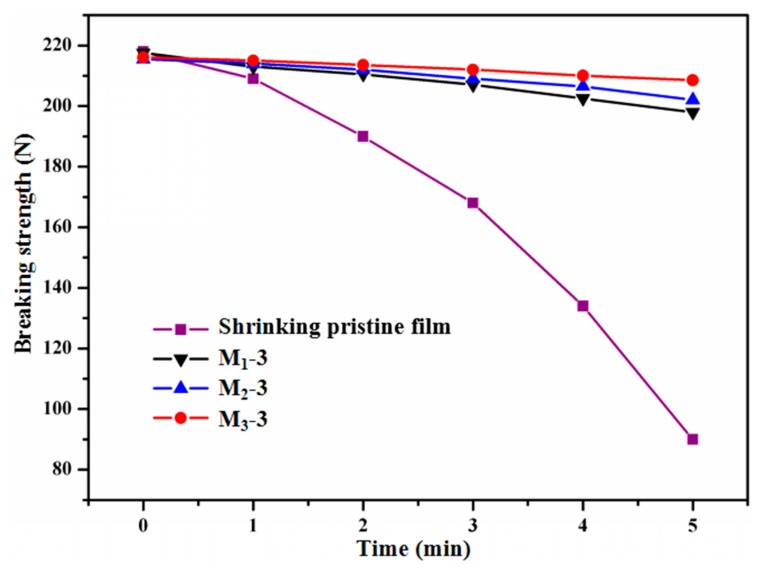
Breaking strength of samples under different UV irradiation times.

Ultraviolet radiation can cause molecular chain breakage and consequently result in deterioration of the mechanical properties of the material [45], thus the changes in breaking strength of materials under UV can reflect its anti-UV property. The breaking strength of shrinking HSPVC films under different UV radiation time is shown in Figure 8. With the increment of UV irradiation time, the breaking strength of the shrinking pristine film decreased rapidly, which indirectly reflects its poor aging resistance. Compared with the shrinking pristine film, the breaking strength of the HSPVC films modified by PTFE nanoparticles deposition decreased slightly, especially that of M_3_-3, which maintained a high breaking strength. When the UV irradiation time was 5 min, the breaking strength of the original HSPVC film decreased by 58.70%, while that of M_1_-3, M_2_-3, and M_3_-3 decreased by only 8.97%, 6.26%, and 3.47%, respectively. The substrate achieves an excellent anti-UV property based on the ability to reflect UV of fluoropolymer [46]. Combined with the surface morphology obtained by SEM, this might be due to the fact that the deposited PTFE coating pretreated with CF_4_ plasma has the fewest pores after shrinkage, which reduces the contact between UV radiation and HSPVC film, thus avoiding the aging of the HSPVC film. The excellent UV resistance of the PTFE/HSPVC composite film is beneficial to extending its outdoor operation life.

### 3.5. Adhesive Strength of PTFE/HSPVC Composite Films

**Figure 9:**
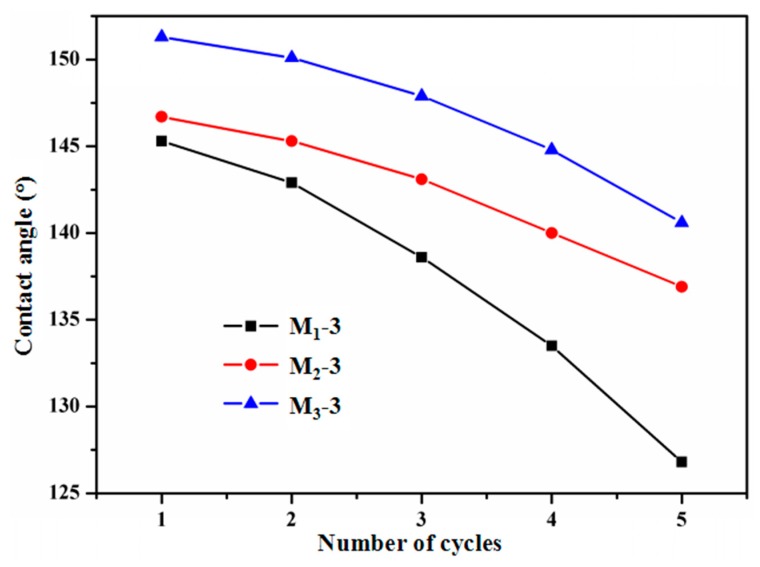
Typical curve of AWCA of PTFE/HSPVC composite films in five cycles of adhesion and stripping between samples and copper foil adhesive tape.

The changes in the AWCA of the modified films shrinking for 60 s subjected to five cycles of adhesion-peel are described in Figure 9. The adhesion fastness between the PTFE coating and substrate was indirectly evaluated by the peel adhesion test. Smaller adhesion fastness of PTFE coating to the substrate is demonstrated by larger AWCA changes. During the five cycles of the peel adhesive process, the AWCA of M_1_-3 decreased significantly from 145° to 127°. Compared with M_1_-3, the AWCA of M_2_-3 and M_3_-3 decreased slightly. It can be inferred that the PTFE coating deposited on the surface of M_1_-3 exhibits the lowest adhesive strength to the substrate. It can be considered in conjunction with Figure 4 that the structure of deposited PTFE coatings without plasma pretreatment is looser and more likely to be destroyed, resulting in poor adhesive strength. Moreover, the AWCA of M_2_-3 decreased by 10°, while M_3_-3 decreased by 11°, suggesting that the PTFE nanoparticles on the surface of M_2_-3 is more difficult to peel off. After five cycles of the peel adhesive process, the AWCA of M_3_-3 remained above 140°. Thus, Ar plasma cleaning provides extremely favorable conditions for the adhesion of PTFE coatings to the substrate, and CF_4_ plasma pretreatment can also enhance the bonding between the PTFE coating and the substrate.

### 3.6. Anti-Icing Property of PTFE/HSPVC Composite Films

**Figure 10:**
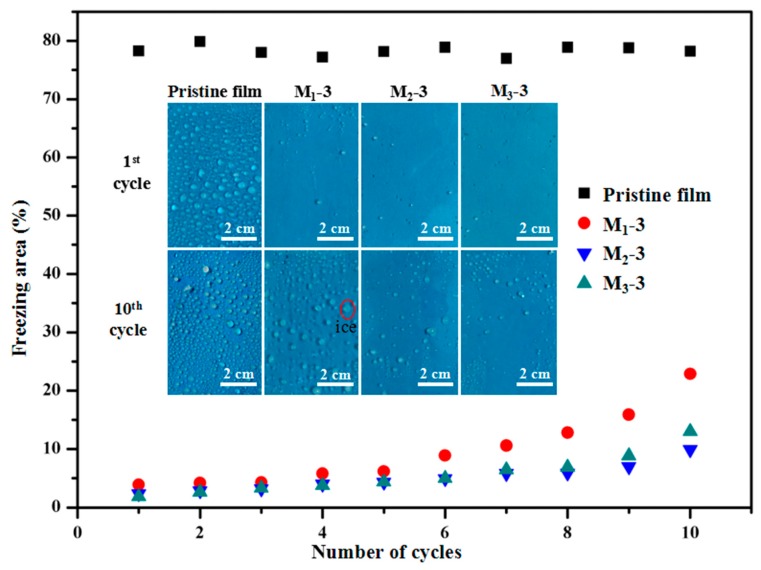
The freezing area ratio on shrinking pristine HSPVC film and shrinking PTFE/HSPVC composite films in the ten cycles of icing and deicing. The illustration corresponds to digital photos of shrinking pristine film, M_1_-3, M_2_-3, and M_3_-3.

It is generally known that excessive accumulation of ice on the blades of wind turbines will seriously affect the working efficiency of wind turbines and even cause the turbines to stop working. The anti-icing property and reusable performance of modified HSPVC film are very important when the blades are coated with modified HSPVC film. Therefore, an artificial climate chamber was used to simulate “freezing rain” to test the anti-icing property of the modified films. It can be seen from Figure 10 that the surface of shrinking HSPVC pristine film was uniformly covered with a large amount of ice with nearly an 80% freezing area ratio, and there was no obvious change in the freezing area ratio during the repeated 10 freezing cycles. The surfaces of PTFE/HSPVC composite films were covered with a little ice, whose freezing rate was greatly reduced compared with the shrinking pristine film. After the first freezing experiment, M_1_-3, M_2_-3, and M_3_-3 all had extremely low freezing area, especially for M_3_-3 pretreated with CF_4_ plasma, whose freezing area ratio was only 1.9%. With the increase of freezing times, the freezing area of the three modified films increased slowly, which might result from the destruction of the PTFE coatings by mechanical deicing. At ten cycles of icing-deicing, the ice resistance of M_1_-3 with a 22.86% freezing area ratio was significantly worse than that of M_2_-3 and M_3_-3. The main reason for the evident increase of freezing area on the surface of M_1_-3 might be the poor adhesive strength between PTFE coating and substrate. Furthermore, the change in freezing area ratio on the PTFE coating deposited on plasma-cleaned HSPVC is relatively small, so it can be speculated that the changes in the microstructure and nanoparticles’ coverage of the PTFE coatings caused by plasma cleaning improve the anti-icing property and recycling times of the PTFE/HSPVC composite films.

**Figure 11:**
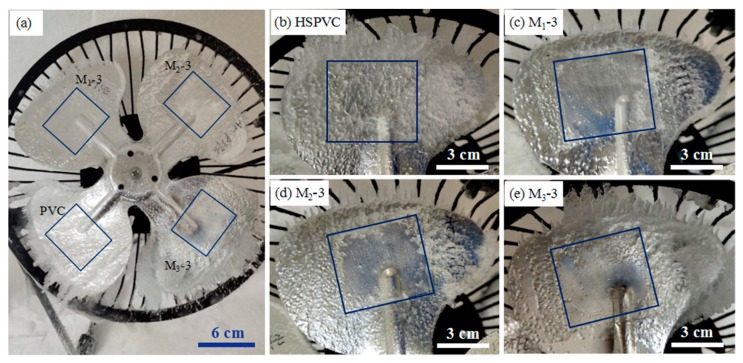
Dynamic icing diagrams of (**a**) electric fan, (**b**) PVC, (**c**) M_1_-3, (**d**) M_2_-3, (**e**) M_3_-3.

Figure 11 depicts the ice accretion on the surface of samples on the electric fan blades over 3 h. Due to the high room temperature, the ice on the blade surface melted gradually after the electric fan was removed from the artificial climate chamber, resulting in slight difference between the image taken and the appearance of the electric fan in the artificial climate chamber. The blue marks on the diagrams represent the size of the samples fixed on the blade. The results showed that a large amount of ice accumulated on the surface of HSPVC membrane after 3 h. On the contrary, only a small amount of ice formed on the edge of the composite film, which might be due to the condensation of “freezing rain” on the surface of the blades and growth to the surface of the composite films, resulting in icing on the edge of the modified films. Obviously, the deposition of the PTFE coating improves the dynamic anti-icing properties of the HSPVC film.

## 4. Conclusions

PTFE/HSPVC composite films were successfully fabricated by depositing PTFE nanoparticles on the surface of HSPVC films via vacuum thermal evaporation of PTFE powders. The effect of different pretreatment conditions on the surface morphology, chemical composition, and surface properties of PTFE/HSPVC composite films was explored, including Ar and CF_4_ plasma cleaning as pretreatment modes, respectively. Compared with other pretreatment methods, the PTFE coating deposited on the HSPVC film using Ar plasma pretreatment is more uniform with a thickness of 4.32 μm. The PTFE nanoparticles on the surface of deposited modified HSPVC films gradually approached uniformity along with the shrinkage of HSPVC films, forming dense PTFE coatings. The AWCA of the PTFE/HSPVC composite film pretreated by CF_4_ plasma can reach 150° and is slightly increased after shrinking, resulting in the formation of superhydrophobic surface. Deposited PTFE coatings contain -CF*_x_* (*x* = 1, 2, 3), and that deposited after CF_4_ plasma pretreatment has the highest F/C elemental ratio. The anti-UV property of the HSPVC films was obviously improved by deposition of PTFE nanoparticles. The peel adhesion test results illustrated that the PTFE coating pretreated by Ar plasma had the highest adhesive strength, while the modified film without plasma pretreatment has the lowest adhesive strength. The PTFE coatings deposited on HSPVC films pretreated by Ar or CF_4_ plasma cleaning has excellent anti-icing property even after 10 cycles of the icing–deicing process.

## Figures and Tables

**Table 1:** Contents of fluorine and carbon in the PTFE coatings with different pretreatment methods.

Pretreatment Methods	Fluorine Content (at %)	Carbon Content (at %)	F/C
Nonplasma cleaning	64.08	33.64	1.91
Ar plasma cleaning	67.47	31.7	2.13
CF_4_ plasma cleaning	68.82	30.54	2.25

## References

[1] Wang Y., Xu Y., Huang Q. (2017). Progress on ultrasonic guided waves de-icing techniques in improving aviation energy efficiency. Renew. Sustain. Energy Rev..

[2] Zuo Z., Liao R., Guo C., Yuan Y., Zhang A. (2015). Fabrication and anti-icing property of coral-like superhydrophobic aluminum surface. Appl. Surf. Sci..

[3] Muhaimin, Kandasamy R., Hashim I. (2010). Effect of chemical reaction, heat and mass transfer on nonlinear boundary layer past a porous shrinking sheet in the presence of suction. Nucl. Eng. Des..

[4] Kondratov A.P., Volinsky A.A., Chen J. (2016). Macro-mechanism of polyvinyl chloride shrink sleeves embossed marking. J. Appl. Polym. Sci..

[5] Chang K.H., Maeng H., Song K., Kaang S. (2009). Thermal behaviors of heat shrinkable poly(vinyl chloride) film. J. Appl. Polym. Sci..

[6] Xu R. (2000). Application of Heat Stabilizer–Methyl Stannum Mercaptide in Poly (vinyl chloride). China Plast..

[7] Alizadeh A., Yamada M., Li R., Shang W., Otta S. (2012). Dynamics of ice nucleation on water repellent surfaces. Langmuir.

[8] Liao R., Zuo Z., Guo C., Zhang A., Zhao X., Yuan Y. (2015). Anti-icing performance in glaze ice of nanostructured film prepared by RF magnetron sputtering. Appl. Surf. Sci..

[9] Gao Q., Hu J., Li R., Pang L., Xing Z., Xu L., Wang M., Guo X., Wu G. (2016). Preparation and characterization of superhydrophobic organic-inorganic hybrid cotton fabrics via γ-radiation-induced graft polymerization. Carbohyd. Polym..

[10] Anna Maria C., Yujun S., Gleason K.K. (2012). Grafted crystalline poly-perfluoroacrylate structures for superhydrophobic and oleophobic functional coatings. Adv. Mater..

[11] Liu X., Ye Q., Song X., Zhu Y., Cao X., Liang Y., Zhou F. (2011). Responsive wetting transition on superhydrophobic surfaces with sparsely grafted polymer brushes. Soft Matter.

[12] Zhang Y.-P., Yang J.-H., Li L.-L., Cui C.-X., Li Y., Liu S.-Q., Zhou X.-M., Qu L.-B. (2019). Facile fabrication of superhydrophobic copper-foam and electrospinning polystyrene fiber for combinational oil–water separation. Polymers.

[13] Liu S., Liu X., Latthe S.S., Gao L., An S., Yoon S.S., Liu B., Xing R. (2015). Self-cleaning transparent superhydrophobic coatings through simple sol–gel processing of fluoroalkylsilane. Appl. Surf. Sci..

[14] Tang Y., Zhang Q., Zhan X., Chen F. (2015). Superhydrophobic and anti-icing properties at overcooled temperature of a fluorinated hybrid surface prepared via a sol-gel process. Soft Matter.

[15] Tripathi S., Haque S.M., Rao K.D., De R., Shripathi T., Deshpande U., Ganesan V., Sahoo N.K. (2016). Investigation of optical and microstructural properties of RF magnetron sputtered PTFE films for hydrophobic applications. Appl. Surf. Sci..

[16] Kylián O., Petr M., Serov A., Solař P., Polonskyi O., Hanuš J., Choukourov A., Biederman H. (2014). Hydrophobic and super-hydrophobic coatings based on nanoparticles overcoated by fluorocarbon plasma polymer. Vacuum.

[17] Bayat A., Ebrahimi M., Nourmohammadi A., Moshfegh A.Z. (2015). Wettability properties of PTFE/ZnO nanorods thin film exhibiting UV-resilient superhydrophobicity. Appl. Surf. Sci..

[18] Park B.J., Furst E.M. (2010). Fabrication of unusual asymmetric colloids at an oil−water interface. Langmuir.

[19] Kim D.-K., Lee S.-B., Doh K.-S. (1998). Surface Properties of Fluorosilicone copolymers and their surface modification effects on PVC film. J. Colloid. Interf. Sci..

[20] Marmur A., Della Volpe C., Siboni S., Amirfazli A., Drelich J.W. (2017). Contact angles and wettability: Towards common and accurate terminology. Surf. Innov..

[21] Marmur A. (2009). A guide to the equilibrium contact angles maze. Contact Angle Wettability and Adhesion.

[22] Wang K., Hou D., Wang J., Wang Z., Tian B., Liang P. (2018). Hydrophilic surface coating on hydrophobic PTFE membrane for robust anti-oil-fouling membrane distillation. Appl. Surf. Sci..

[23] Huang Q., Huang Y., Gao S., Zhang M., Xiao C. (2018). Novel Ultrafine Fibrous Poly(tetrafluoroethylene) hollow fiber membrane fabricated by electrospinning. Polymers.

[24] Jiang Z., Jiang L., Jia H., Zhou Y., Ma J., Chen S. (2018). Modification of polytetrafluoroethylene-fiberglass composite film using polydopamine deposition with improved hydrophilicity. Fiber Polym..

[25] Prelipceanu M., Tudose O.-G., Prelipceanu O.-S., Schrader S., Grytsenko K. (2007). Study of oriented growth of oligofluorene–thiophene films onto aligned vacuum-deposited polytetrafluoroethylene layers. Mat. Sci. Semicon. Proc..

[26] Kim H.-M., Sohn S., Ahn J.S. (2013). Transparent and super-hydrophobic properties of PTFE films coated on glass substrate using RF-magnetron sputtering and Cat-CVD methods. Surf. Coat. Technol..

[27] Zhan Y.L., Ruan M., Li W., Li H., Hu L.Y., Ma F.M., Yu Z.L., Feng W. (2017). Fabrication of anisotropic PTFE superhydrophobic surfaces using laser microprocessing and their self-cleaning and anti-icing behavior. Colloid. Surf. A.

[28] Zhuang A., Liao R., Dixon S.C., Lu Y., Sathasivam S., Parkin I.P., Carmalt C.J. (2017). Transparent superhydrophobic PTFE films via one-step aerosol assisted chemical vapor deposition. RSC Adv..

[29] Alawajji R.A., Kannarpady G.K., Biris A.S. (2018). Fabrication of transparent superhydrophobic polytetrafluoroethylene coating. Appl. Surf. Sci..

[30] Yi N., Bao S., Zhou H., Xin Y., Huang A., Ma Y., Li R., Jin P. (2016). Preparation of microstructure-controllable superhydrophobic polytetrafluoroethylene porous thin film by vacuum thermal-evaporation. Front. Mater. Sci..

[31] Wang J.M., Wang L.D., Feng L. (2011). One-step fabrication of fluoropolymer transparent films with superhydrophobicity by dry method. J. Appl. Polym. Sci..

[32] Li S.-Y., Li Y., Wang J., Nan Y.-G., Ma B.-H., Liu Z.-L., Gu J.-X. (2016). Fabrication of pinecone-like structure superhydrophobic surface on titanium substrate and its self-cleaning property. Chem. Eng. J..

[33] Audic J.-L., Poncin F., Reyx D., Brosse J.-C. (2001). Cold plasma surface modification of conventionally and nonconventionally plasticized poly(vinyl chloride)-based flexible films: Global and specific migration of additives into isooctane. J. Appl. Polym. Sci..

[34] Sohbatzadeh F., Mirzanejhad S., Ghasemi M., Talebzadeh M. (2013). Characterization of a non-thermal plasma torch in streamer mode and its effect on polyvinyl chloride and silicone rubber surfaces. J. Electrostat..

[35] Tan C., Cai P., Xu L., Yang N., Xi Z., Li Q. (2015). Fabrication of superhydrophobic surface with controlled adhesion by designing heterogeneous chemical composition. Appl. Surf. Sci..

[36] Kamegawa T., Shimizu Y., Yamashita H. (2012). Superhydrophobic surfaces with photocatalytic self-cleaning properties by nanocomposite coating of TiO(2) and polytetrafluoroethylene. Adv. Mater..

[37] Ru L., Jie-rong C. (2006). Studies on wettability of medical poly(vinyl chloride) by remote argon plasma. Appl. Surf. Sci..

[38] Asadinezhad A., Lehocký M., Sáha P., Mozetič M. (2012). Recent progress in surface modification of polyvinyl chloride. Materials.

[39] Wen X.Q., Liu X.H., Liu G.S. (2010). Improvement in the hydrophilic property of inner surface of polyvinyl chloride tube by DC glow discharge plasma. Vacuum.

[40] Fresnais J., Chapel J.P., Poncin-Epaillard F. (2006). Synthesis of transparent superhydrophobic polyethylene surfaces. Surf. Coat. Technol..

[41] Wei X., Zhao B., Li X.M., Wang Z., He B.Q., He T., Jiang B. (2012). CF_4_ plasma surface modification of asymmetric hydrophilic polyethersulfone membranes for direct contact membrane distillation. J. Membr. Sci..

[42] Tian M., Yin Y., Yang C., Zhao B., Song J., Liu J., Li X.-M., He T. (2015). CF_4_ plasma modified highly interconnective porous polysulfone membranes for direct contact membrane distillation (DCMD). Desalination.

[43] Dreux F., Marais S., Poncin-Epaillard F., Métayer M., Labbé M. (2002). Surface modification by low-pressure plasma of polyamide 12 (PA12). improvement of the water barrier properties. Langmuir.

[44] Park S.-J., Sohn H.-J., Hong S.-K., Shin G.-S. (2009). Influence of atmospheric fluorine plasma treatment on thermal and dielectric properties of polyimide film. J. Colloid. Interf. Sci..

[45] Wang B., Duan Y., Zhang J. (2016). Titanium dioxide nanoparticles-coated aramid fiber showing enhanced interfacial strength and UV resistance properties. Mater. Des..

[46] Gotoh K., Nakata Y., Tagawa M., Tagawa M. (2003). Wettability of ultraviolet excimer-exposed PE, PI and PTFE films determined by the contact angle measurements. Colloid. Surf. A.

